# SG-APSIC1118: Introduction of carbapenemase-producing Enterobacterales (CPE) in the aqueous environment of the newly built National Centre for Infectious Diseases (NCID) in Singapore

**DOI:** 10.1017/ash.2023.36

**Published:** 2023-03-16

**Authors:** Chong Hui Clara Ong, Kyaw Zaw Linn, Sharifah Farhanah, Huan Xiaowei, Liang Hui Loo, Danielle Hui Ru Tan, Nur Hafizah Binte Hamad, Allie Yin Lim, Chu Ying Poon, Ying Wei Tang, Pei Yun Hon, Shawn Vasoo, Oon Tek Ng, Kalisvar Marimuthu

**Affiliations:** National Centre for Infectious Diseases, Singapore; National Public Health & Epidemiology Unit, National Centre for Infectious Diseases, Singapore; Infectious Disease Research and Training Office, National Centre for Infectious Diseases, Singapore; Infectious Disease Research and Training Office, National Centre for Infectious Diseases, Singapore; Infectious Disease Research and Training Office, National Centre for Infectious Diseases, Singapore; National Public Health & Epidemiology Unit, National Centre for Infectious Diseases, Singapore; National Public Health & Epidemiology Unit, National Centre for Infectious Diseases, Singapore; National Public Health & Epidemiology Unit, National Centre for Infectious Diseases, Singapore; National Public Health & Epidemiology Unit, National Centre for Infectious Diseases, Singapore; National Public Health & Epidemiology Unit, National Centre for Infectious Diseases, Singapore; Infectious Disease Research and Training Office, National Centre for Infectious Diseases, Singapore; Infectious Diseases, Tan Tock Seng Hospital, Singapore; Infectious Diseases, Tan Tock Seng Hospital, Singapore; Infectious Diseases, Tan Tock Seng Hospital, Singapore

## Abstract

**Objectives:** In healthcare facilities, environmental reservoirs of CPE are associated with CPE outbreaks. In the newly built NCID building, we studied the introduction of CPE in the aqueous environment. **Methods:** We sampled the aqueous environments (ie, sink, sink strainer, and shower drain-trap with Copan E-swabs and sink P-trap water) of 4 NCID wards (ie, 2 multidrug-resistant organism (MDRO) wards and 2 non-MDRO wards). Two sampling cycles (cycle 1, June–July 2019 and cycle 2, September–November 2019) were conducted in all 4 wards. Cycle 3 (November 2020) was conducted in 1 non-MDRO ward to investigate CPE colonization from previous cycles. Enterobacterales were identified using MALDI-TOF MS and underwent phenotypic (mCIM and eCIM) and confirmatory PCR tests for CPE. **Results:** We collected 448, 636, and 96 samples in cycles 1, 2, and 3, respectively. MDRO and non-MDRO wards were operational for 1 and 7 months during the first sampling cycle. The CPE prevalence rates in MDRO wards were 1.67% (95% CI, 0.46% – 4.21%) in cycle 1 and 1.76% (95% CI, 0.65% – 3.80%) in cycle 2. In the aqueous environments in MDRO wards, multiple species were detected (cycle 1: 2 *K. pneumoniae*, 1 *E. coli*, and 1 *S. marcescens*; cycle 2: 5 *K. pneumoniae* and 1 *R. planticola*), and multiple genotypes were detected (cycle 1: 3 *bla*OXA48; cycle 2: 5 *bla*OXA48 and 1 *bla*KPC). The CPE prevalence in non-MDRO wards was 1.92% (95% CI, 0.53%–4.85%) in cycle 1. The prevalence rate increased by 5.51% (95% CI, 1.99%–9.03%) to 7.43% (95% CI, 4.72%–11.04%; *P* = .006) in cycle 2, and by another 2.98% (95% CI, −3.82% to 9.79%) to 10.42% (95% CI, 5.11% – 18.3%; *P* = .353) in cycle 3. Only *bla*OXA48 *S. marcescens* were detected in all cycles (except 1 *bla*OXA48 *K. pneumoniae* in cycle 2) in the non-MDRO ward. **Conclusions:** CPE established rapidly in the aqueous environment of NCID wards, more so in MDRO wards than non-MDRO wards. Longitudinal studies to understand the further expansion of the CPE colonization and its impact on patients are needed.

